# Effects of Twitter use on academic performance and satisfaction in a pathophysiology course among Omani nursing students: a quasi-experimental study

**DOI:** 10.1186/s12912-023-01609-x

**Published:** 2023-11-21

**Authors:** Mickaël Antoine Joseph, Jansirani Natarajan, Vidya Seshan, Erna Judith Roach, Omar Al Omari, Suja Karkada

**Affiliations:** 1 Fundamentals and Administration Department, College of Nursing, Sultan Qaboos University, Muscat, Oman; 2 Maternal and Child Health Department, College of Nursing, Sultan Qaboos University, Muscat, Oman

**Keywords:** Students nursing, Twitter, Education, Academic performance, Satisfaction, Oman

## Abstract

**Background:**

Nursing students often find bioscience courses, such as pathophysiology, challenging. Utilizing Twitter to provide concise course content and answer students’ questions before exams may be beneficial. The objective of this study was to determine if using Twitter can improve nursing students’ academic performance and satisfaction with pathophysiology courses.

**Methods:**

A post-test, two-group quasi-experimental research design was employed in this study. It involved second-year Bachelor of Nursing students participating in a pathophysiology course at the College of Nursing, Sultan Qaboos University, in Muscat, Oman. Seventy-three second-year Bachelor of Nursing students participated in the pathophysiology course; 50 students opted to use Twitter, forming the experimental group, while the remaining 23, who chose not to use Twitter, formed the control group. We used Twitter to provide concise course content for the pathophysiology course and conduct one-hour question-and-answer sessions the night before exams. Academic performance was assessed through examination scores, and student satisfaction levels with Twitter was measured using five-point Likert scale questionnaires. Data were analyzed using Mann-Whitney and t-tests.

**Results:**

Although there was no significant difference in final exam scores between the experimental and control groups, survey results showed that students were generally satisfied with the incorporation of Twitter in the pathophysiology course, including the question-and-answer sessions.

**Conclusions:**

The findings suggest that Twitter can serve as a valuable tool for enhancing nursing student satisfaction with the pathophysiology course.

**Supplementary Information:**

The online version contains supplementary material available at 10.1186/s12912-023-01609-x.

## Background

Pathophysiology is crucial for nursing students, as it equips them with the necessary skills to critically analyze clinical cases and ensure safe practice [1]. Success in pathophysiology predicts students’ success in nursing certification examinations [2]. However, as a bioscience course, pathophysiology is often perceived as one of the least engaging and most challenging courses in the nursing curriculum, both for students to learn and for nursing faculty members to teach [3, 4]. Employing innovative teaching strategies can help reduce the course’s complexity while enhancing academic achievement and satisfaction among nursing students [5].

Social media is widely used by undergraduate nursing students [6]. VJA Duke, A Anstey, S Carter, N Gosse, KM Hutchens and JA Marsh [7] found that most undergraduate nursing students utilize social media for educational purposes. The use of social media among nursing students has been acknowledged as valuable for education [8], despite some caution surrounding its use [9]. One of the primary concerns is the potential for distraction, as students may engage with non-educational content. Misinformation is another critical issue, as the unregulated nature of social media can lead to the spread of inaccurate or misleading information, particularly detrimental in healthcare education [10]. Privacy concerns are also important, as sharing personal or patient information can lead to breaches of confidentiality and professional conduct [11]. Additionally, the risk of unprofessional behavior on these platforms can have serious implications for nursing students, potentially impacting their future careers and the reputation of their educational institutions [12].

Twitter, which has recently been renamed to ‘X’, has become popular among nursing students and registered nurses to connect online with nursing communities and for use in undergraduate nursing programs at conferences [13]. Twitter is a microblogging social media platform that allows users to post messages of up to 280 characters, along with features such as hashtags, likes, replies, retweets, and mentions [14].

Few studies have explored the use of Twitter in nursing education. R Jones, J Kelsey, P Nelmes, N Chinn, T Chinn and T Proctor-Childs [15] used Twitter to enhance the digital professionalism of first-year nursing students. Students perceived Twitter as valuable for enhancing peer support and promoting a better understanding of social media. They also considered Twitter an effective platform for learning about student life and interesting health topics. Similarly, TM Stephens and ME Gunther [16] used Twitter to deliver information and ask questions about a resilience program, finding it to be a convenient and enjoyable tool for disseminating educational messages to undergraduate nursing students. AM Price, K Devis, G LeMoine, S Crouch, N South and R Hossain [17] used Twitter as an engaging teaching tool in the undergraduate nursing curriculum, specifically to discuss a first-year module on nursing. This approach enabled students to communicate with one another, share ideas, broaden their global perspectives, and increase awareness of nursing issues. EA Gazza [18] used Twitter to engage students in healthcare policy initiatives, with students following Twitter accounts related to healthcare policy and found the experience eye-opening and helpful for staying up-to-date. However, to the best of our knowledge, no studies have yet used Twitter as a learning tool in a nursing bioscience course, such as pathophysiology, to present course material and answer students’ questions in real time.

Twitter’s value lies in its ability to provide near-instant feedback and convey information concisely with its 280-character limit. When used to promote group discussions among qualified and pre-registration nurses, the brevity of tweets was beneficial in helping students summarize and clarify their understanding of course material [19]. Moreover, Twitter has been shown to enhance the contact between faculty and students, providing a platform for immediate feedback [20]. SM Gonzalez and CC Gadbury-Amyot [21] discussed the benefits of providing students at dental school with swift responses to their exam questions, ensuring that students save time searching for answers when time is limited before exams or avoiding the development of incorrect thoughts about the course content. These authors found that the concise nature of Twitter messages makes it an ideal platform for students to ask focused questions and receive direct and succinct responses from the instructor. However, this study did not examine the impact of Twitter use on students’ academic performance.

Educators have recognized the potential benefits of incorporating Twitter into nursing education, using it to support instruction [17], facilitate course evaluation [22], and connect students with global nursing organizations [18]. However, the satisfaction levels of nursing students using Twitter have not yet been thoroughly investigated.

Therefore, this study aimed to assess the potential benefits of using Twitter in a pathophysiology course for undergraduate nursing students. We chose to utilize Twitter outside of classroom hours due to evidence from previous studies suggesting that using Twitter during class can distract nursing students [17, 23]. Specifically, we aimed to evaluate whether using Twitter to deliver course content concisely and provide a one-hour question-and-answer session the night before exams could improve students’ academic performance and satisfaction with the course.

## Methods

### Study design and participants

This study employed a quasi-experimental, two-group post-test research design. The participants were second-year Bachelor of Nursing students from the College of Nursing at Sultan Qaboos University in Oman, all of whom were Omani nationals. They were enrolled in the pathophysiology course. Pathophysiology is a 4-credit, 5-contact hour course that requires Anatomy and Physiology 2 as a pre-requisite. The course provides an overview of major diseases affecting the 11 body systems. It was delivered in a traditional, face-to-face format during the Spring 2019 semester, prior to the COVID-19 pandemic. Seventy-three students participated in this study: 50 decided to use Twitter as an additional tool in this course, and 23 chose not to use Twitter.

### Use of twitter in the pathophysiology course

The instructor, who is also the study’s first author, created a new Twitter account specifically for teaching the pathophysiology course with the handle: @DrMickaelJosep1.

During the orientation week, students were informed about the Twitter account and encouraged to follow the instructor’s account. All students reported having a smartphone and the ability to download the Twitter application. For those students who did not already have a Twitter account, instructions were provided on how to create one. The Twitter account was set to private to ensure student privacy, and students were required to send their name and ID number to the instructor through a direct message to be admitted to the group. In this way, the instructor knew who was following the account.

Students were provided with the account name (@DrMickaelJosep1) and instructed on how to ask questions on Twitter by including the account name in their tweets. Students were also advised to enable push notifications for tweets from the instructor’s account to receive notifications, similar to a standard text message, whenever the instructor tweeted. Once they entered and viewed the tweet, the notification would disappear, and they could engage with the tweet at their convenience. Additionally, a hashtag (#NURS2019), which is the course code, was created to make it easier for students to find and follow the questions and answers related to the course. Students were advised to use the hashtag in all their tweets. Using the hashtag was important because it allowed students to easily search for and access all of the tweets related to the course. It was emphasized to the students that using Twitter was voluntary and that it was not a requirement of the course. They were informed that not using Twitter would not have a negative impact on their participation in the course in any way.

Starting from the second week, the instructor delivered approximately three tweets per day for one week, and then there were no tweets the following week. The reason for not delivering tweets every week was to prevent students from feeling overwhelmed with too much information and give them time to process the previous week’ information effectively. The tweets aligned with the course outline and covered similar content to what was covered in class each two weeks. This helped reinforce the material taught in class and provided students with additional resources for learning and studying. The tweets included concise and objective facts about pathophysiology, questions for discussion, images, infographics, and polls for students to answer (e.g., “What is a normal process of cell death in tissues also known as ‘cellular suicide’? [options: apoptosis, necrosis, autophagy, accumulation]”).

Students were able to interact with the tweets in various ways, including liking a tweet, retweeting it on their own profile, or commenting on the tweet. For the polls, students could also select an option. The content of the tweets was guided by the course learning objectives and was based on the textbook used for teaching, “Gould’s Pathophysiology for the Health Professions, 6th Edition.“ The objectives of the tweets were as follows:

- Reinforcing important information (e.g., “The buffer ratio of 20 parts bicarbonate ion (base) to one part CO_2_ (carbonic acid) is essential to maintain serum pH in the normal range of 7.35 to 7.45”).
- Stimulating critical thinking (e.g., “Aldosterone causes retention of sodium and water. Why do you think Addison’s disease causes hyponatremia?“).
- Enhancing communication (e.g., “Mr. Omar has left ventricular hypertrophy from hypertension. ‘How does that increase my risk of heart attack?‘ he asks. ‘I thought big, strong muscles were a good thing?‘ How would you answer him?“).
- Encouraging students to seek out additional information (e.g., “Check out this article about different types of necrosis: liquefactive, coagulative, caseous, fat, fibrinoid, and gangrenous. https://www.ncbi.nlm.nih.gov/books/NBK430935/”).

### Question-and-answer sessions before the exams

Additionally, the instructor held one-hour question and answer (Q&A) sessions on Twitter prior to the first, second, and final exams. During these sessions, students were able to post questions and receive instant answers from the instructor. Students were instructed to ask their questions on Twitter using either the hashtag #NURS2019 or by including the handle of the course coordinator (@DrMickaelJosep1) in their tweet, or by sending a direct message to the course coordinator. The instructor would retweet the student’s question from his account, without including the student’s name, and then tweet the answer for all students to see. This provided students with an opportunity to ask questions the night before the exams and receive clarification in a timely manner.

### Data collection procedure

The pathophysiology course consisted of three major exams: the first in-course exam (30%), the second in-course exam (30%), and the final exam (40%). After each exam, the instructor collected students’ performance data, and a final course grade was generated for all students. The final course grades were then compared between the experimental group (students who used Twitter) and the control group (students who chose not to use Twitter).

To assess students’ use of Twitter and satisfaction with using Twitter in the course, a survey was developed and administered to students after they completed their final examination (week 16). The survey aimed to gather information about students’ experiences and satisfaction with using Twitter in the pathophysiology course. The instructor explained the objectives of the survey and emphasized that participation was completely voluntary. To ensure privacy, the instructor then left the room so that students could complete the survey without any distractions.

The survey consisted of several sections including (1) demographic information, (2) students’ use of Twitter, and satisfaction with (3) the question-and-answer sessions and (4) the use of Twitter in the pathophysiology course. The survey also included (5) two open-ended questions, which allowed students to provide more in-depth and personalized feedback about their experiences using Twitter in the pathophysiology course. The first open-ended question asked students to describe what they liked about using Twitter in the course, while the second asked them to describe what they did not like (The questionnaire is available in the supplementary material).

### Students’ satisfaction with the use of twitter

Students’ satisfaction with Twitter as a teaching tool was evaluated using two validated and reliable questionnaires. Only students who used Twitter in the course were asked to complete these two questionnaires.

The first questionnaire consisted of six items and was used to evaluate students’ perceptions of the question-and-answer sessions on Twitter. It was adapted from a questionnaire developed by Gonzalez and Gadbury-Amyot (2016) [21] with their approval and was found to be reliable in this study, with a Cronbach’s alpha of 0.832.

The second questionnaire consisted of five items and evaluated students’ general satisfaction with the use of Twitter in the pathophysiology course. It was adapted from a similar tool developed by Lowe and Laffey (2011) [24] with their approval and had a high reliability in their study (0.981). In this study, Cronbach’s alpha was also high (α = 0.894).

Both questionnaires used a 5-point Likert scale, ranging from 1 (strongly disagree) to 5 (strongly agree).

### Data analysis

In this study, data analysis was performed using IBM SPSS version 23. Descriptive statistics were used to summarize the demographic characteristics and the use of Twitter for the pathophysiology course. ANOVA (Analysis of Variance) and Chi-Square were used to assess potential differences between the control and experimental groups in terms of demographic characteristics and prior use of Twitter. Mann-Whitney U test was applied to compare the academic performance of students who used Twitter and those who did not. Lastly, a series of t-tests were conducted to evaluate the satisfaction levels of students using Twitter for question-and-answer sessions and overall use in the Pathophysiology course. The one sample t-tests were used to compare the mean satisfaction scores of the students who used Twitter for question-and-answer sessions and for overall use to a hypothetical value of 3, which represents a neutral response on the five-point Likert scale. This allowed us to determine if the satisfaction levels were significantly different from neutral.

### Ethical considerations

The study was conducted in accordance with ethical standards and was approved by the ethics committee at Sultan Qaboos University College of Nursing in Oman (CON//EA/22/2019). Participation in Twitter use was voluntary, and students had the choice to opt in or out, which lead to the formation of a control group (although smaller than the experimental group). All students provided written informed consent before completing the survey.

## Results

### Participant characteristics

**Fig. 1:**
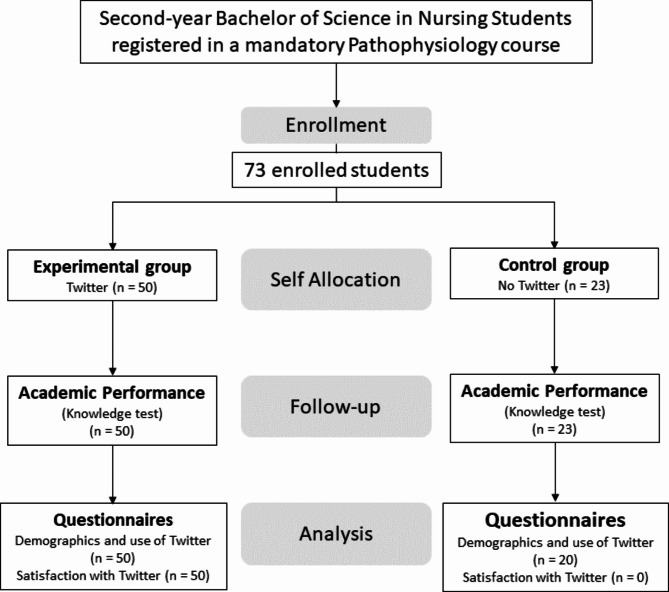
Study Flowchart

Of the 73 students enrolled in the pathophysiology course in the Spring of 2019 semester, 50 students chose to use Twitter and formed the experimental group, while 23 decided not to and formed the control group. Figure 1 illustrates a comprehensive flow diagram for the study.

The experimental group comprised 44 female and 6 male students, with an average age of 23.02 years and a standard deviation of 3.89. The control group consisted of 15 female and 8 male students, with an average age of 22.26 years and a standard deviation of 3.79.

Most students in both groups were familiar with Twitter prior to its use in the pathophysiology course. However, 72% of the students in the experimental group who used Twitter for the course already had a Twitter account, compared to only 30% of those in the control group who had a Twitter account. This was the only statistically significant difference in demographic characteristics between the two groups, as shown in Table 1.

**Table 1:** Demographic Characteristics and the use of Twitter for pathophysiology course (n = 70)

		Used Twitter (n = 50)	Did not use Twitter (n = 20)	ANOVA F	P value
Age (years ± SD)		23.02 ± 3.89	22.26 ± 3.79	0.519	0.474
cGPA (mean ± SD)		3.01 ± 0.37	3.11 ± 0.38	0.469	0.498
		n (%)	n (%)	Chi Square	P value
Gender	Female	44 (88.0)	15 (75.0)	1.823	0.177
	Male	6 (12.0)	5 (25.0)		
Before this year, have you heard of twitter?	Yes	48 (96.0)	18 (90.0)	0.955	0.931
	No	2 (4.0)	2 (10.0)		
Did you have a Twitter account prior to this year?	Yes	36 (72.0)	6 (30.0)	10.500	0.001
	No	14 (28.0)	14 (70.0)		

cGPA: Cumulative Grade Point Average ; SD : Standard Deviation

### Twitter usage

Over the course of the 15-week Spring 2019 semester, a total of 181 tweets were generated. On average, 30 questions were asked and answered during the one-hour question-and-answer sessions held the night before each exam.

### Students’ academic performance

The students’ final scores in the pathophysiology course did not show a significant difference between the experimental group (mean = 76.99, SD = 10.02, n = 50) and the control group (mean = 76.77, SD = 8.45, n = 23). The results of the statistical analysis (U = 546.5, z = -0.338, *p* = 0.735) indicate no significant difference. The data in Table 2 confirms the lack of statistically significant differences between the two groups.

**Table 2:** Academic performance of students who used Twitter and those who did not use Twitter for pathophysiology course (n = 73)

	Mean ± SD.	U^a^	Z	Sig.
Used Twitter (n = 50)	76.99 ± 10.02	546.5	-0.338	0.735
Did not use Twitter (n = 23)	76.77 ± 8.45			

SD : Standard Deviation

^a^ Mann-Whitney U test

### Students’ satisfaction with the use of twitter

The results of the survey show that students are satisfied with the use of Twitter for question-and-answer sessions, with a mean score of 4.12 on a 5-point Likert scale. Students believed that the sessions on Twitter improved accessibility to the instructor, with the same mean score of 4.12. On the other hand, students were moderately satisfied with the potential positive effect of these sessions on their overall grades, with a mean score of 3.48 (Table 3).

Regarding the general satisfaction with the use of Twitter for the pathophysiology course, nursing students thought that using Twitter was enjoyable (mean score of 4.12) and increased their level of satisfaction with the course (mean score of 3.54). However, they did not find that using Twitter was more enjoyable than listening to a lecture (mean score of 2.67) or more productive (mean score of 2.44) (Table 3).

**Table 3:** Satisfaction with the use of Twitter for the Question-and-Answer Sessions and with the overall use of Twitter for Pathophysiology course (N = 50)

	Mean	SD	t^b^	Sig.
**Satisfaction with the use of Twitter for the Question-and-Answer sessions**				
1. The question-and-answer sessions on Twitter were very helpful	4.12	0.79	9.912	0.000
2. The Twitter question-and-answer sessions improved accessibility to the instructor	4.12	0.89	8.845	0.000
3. In the future I would enjoy using Twitter in other courses	3.74	0.96	5.423	0.000
4. The use of Twitter for question-and-answer sessions had a positive effect on my overall grade	3.52	0.99	3.697	0.001
5. In the future I would enjoy Twitter in the classroom for asking questions during lecture	3.52	1.28	2.869	0.006
6. I feel using Twitter for question-and-answer sessions improved my overall grade	3.48	0.95	3.562	0.001
**Satisfaction with the use of Twitter for the Pathophysiology course**				
1. I believe that using web technologies such as Twitter is enjoyable	4.12	0.69	42.27	0.000
2. Using Twitter increased my overall satisfaction with the course	3.54	0.91	27.56	0.000
3. Using Twitter was one of the best parts of this course	3.48	1.16	21.126	0.000
4. As a learning experience, using Twitter was more enjoyable than listening to a lecture	2.68	1.19	15.98	0.000
5. As a learning experience, using Twitter was more productive than listening to a lecture	2.44	1.13	15.299	0.000

Values are expressed as mean and standard deviation (SD)

^b^ One sample t-test

### Qualitative results

At the end of the survey, 57% of students left additional feedback on using Twitter in the pathophysiology course. The comments were analyzed and found to be mostly positive (22 out of 28). Students praised the benefits of question-and-answer sessions in clarifying confusion and providing a platform for shy students to ask questions. Some reported that Twitter provided more information than lectures and easier communication with the instructor. Negative comments cited concerns over internet speed, information overload before exams, and the preference to concentrate in the classroom. Twelve students in the control group who chose not to use twitter mentioned that they prefer not to use social media, some favoring Instagram and expressed privacy concerns and lack of time.

## Discussion

This study showed that using Twitter as a platform to deliver course content in the pathophysiology course and to provide an immediate response to students’ questions before exams, improved Omani nursing students’ course satisfaction and enjoyment. However, it did not significantly impact their academic performance, nor did they find Twitter more productive or enjoyable than traditional lectures.

We chose Twitter for this study since it has been the most commonly used social media platform for medical education, along with Facebook, from 2017 until now [25]. Despite that, one-third of the students (32%) decided not to use Twitter. Initially, we assumed that all students would be interested in using a social media platform for learning, but this was not the case. Some students had concerns about privacy issues related to the use of Twitter. This is understandable, as previous literature has reported instances of unprofessional behavior and breaches of confidentiality and privacy that have occurred alongside the growth of Twitter [25, 26].

Despite our efforts to mitigate these concerns by making the Twitter account private and requiring students to provide their identification number and name before assessing the account, some students still expressed fear of privacy issues. Therefore, it is essential to develop clear guidelines that outline the responsible use of Twitter during the course and the consequences of misconduct. Students should also be encouraged to use social media to develop digital professionalism skills, which are becoming increasingly important in today’s society. R Booth and S Connor [27] have emphasized that failing to incorporate social media in education is a missed opportunity.

Based on the demographic data, it is evident that most students who did not use Twitter did not initially have a Twitter account. Qualitative data further indicates that these students either have no interest in social media or prefer using a different platform, such as Instagram. Understanding students’ preferences before incorporating social media into the classroom is crucial to ensure maximum student use. Therefore, we recommend that educators gather information on students’ preferred social media platforms at the beginning of the semester and choose the most suitable one accordingly.

Our findings showed no significant difference in academic performance between students who used Twitter and those who did not. Our initial hypothesis was that Twitter usage would improve students’ academic performance by providing more access to course information, content, discussions, polls, and instant answers to students’ questions before exams. Previous studies in nursing education supported the idea that using Twitter can improve student knowledge. However, these studies have shown that Twitter provides additional information on international health, health issues, and nursing topics, enhancing students’ general knowledge rather than improving academic performance [15, 17]. In nursing education literature, Twitter is often used for personal development and for continuous education in conjunction with traditional teaching strategies [25], rather than to improve academic performance. One possible explanation for our results could be the limited character capability of Twitter. While the 280-character limit has been shown to encourage active learning and concise communication of ideas [28], it may have hindered students’ ability to participate in more extensive discussions. Hence, we recommend using Twitter to provide additional information, current debates, and international nursing issues, to enhance students’ clinical reflections and critical thinking skills.

Our results also showed that students had positive views regarding using Twitter and found the question-and-answer sessions helpful, which aligns with previous studies that demonstrated that instant feedback correlates with increased confidence and motivation levels among students [17]. Furthermore, TM Stephens and ME Gunther [16] argued that millennials require instantaneous feedback, and are less likely to participate in discussions if they do not receive it. In that regard, using Twitter in nursing education has improved communication and collaboration among students [25].

Overall, students were also satisfied with using Twitter in the pathophysiology course and found it enjoyable. Studies in medical and dental education also support our findings, showing that students reported higher satisfaction and engagement levels using Twitter in their courses [20, 21]. However, our students did not believe Twitter was more productive or enjoyable than traditional lectures. Therefore, we recommend that nursing educators use Twitter to increase students’ accessibility to the instructor, which may increase their satisfaction with the course.

Finally, our qualitative data demonstrated that students were satisfied with using Twitter, as it offers a platform for shy individuals to ask questions. This finding aligns with the K Gagnon [28] study, which revealed that all class members could participate in online discussions using Twitter, not just those who engage in in-class discussions. It has been shown that online discussions benefit students who are uncomfortable speaking in the classroom by reducing their stress and anxiety levels and empowering them to take charge of their learning [29]. However, one of the negative comments mentioned by our students in this study was that using Twitter before exams created content overload. Similarly, S Warshawski, O Bar-Lev and S Barnoy [30] found that exposing Arab and Israeli students to new knowledge before exams increased their stress and anxiety levels. However, N Scott and D Goode [25] suggested that these results are specific to certain cultures and have yet to be demonstrated elsewhere. Furthermore, students who did not use Twitter stated that a lack of time was one of the main reasons for their decision. This finding is supported by AM Price, K Devis, G LeMoine, S Crouch, N South and R Hossain [17], who reported that social media is perceived as a new skill to be learned and adds to the students’ already heavy workload and stress.

### Limitation

Our study was conducted with a limited sample size and in a single setting, which limits the generalizability of the results. Moreover, the difference in sample size between the experimental and the control groups may have prevented the detection of actual differences in this study. For ethical reasons, we allowed all students to follow the Twitter account, knowing that any additional teaching strategy is beneficial, and we could not deny this opportunity to some students. As a result, two-thirds of the students joined Twitter, while one-third did not, constituting the control group. This led to an imbalanced distribution between the experimental and control groups, which could have impacted our results. Additionally, following the Twitter account does not guarantee interaction with or viewing the tweets. Collecting Twitter analytic data would allow for a more accurate measurement of online engagement. Unfortunately, due to country policies, the Twitter analytics feature was not accessible for our Twitter account. Future studies could make Twitter use a course requirement and allocate a portion of the grade to it, which might encourage students to engage with the tweets and reap more benefits.

It is also important to note that our study only looked at academic performance and satisfaction; however, Twitter is known to have many other benefits, such as promoting communication and collaboration, fostering peer-teaching, increasing confidence, and improving psychological well-being by reducing professional isolation [25]. Therefore, these variables should be investigated when studying the use of Twitter in nursing education.

### Conclusion

Twitter use has significantly increased in recent years, and it has become a common platform for communication, collaboration, and showcasing innovative clinical and educational material [31]. In our study, we used Twitter to deliver course content and provided students with the opportunity to interact with it, and receive instant responses to their questions before the exams. While we found that Twitter did not enhance students’ academic performance, they were satisfied with its use throughout the course and in answering questions before exams.

### Electronic supplementary material

Below is the link to the electronic supplementary material.

Supplementary Material 1

## Data Availability

The datasets used and/or analysed during the current study are available from the corresponding author on reasonable request.

## References

[1] Donkin R, Hatje E, Reinke NB (2022). An eLearning module is comparable to face-to-face teaching in a nursing human pathophysiology subject. Nurse Educ Today.

[2] Silbert-Flagg J, Adams TK, Fava-Hochuli J, Budhathoki C, Jordan E (2018). Results of a study on nursing students’ success in taking advanced level (graduate) pathophysiology in their basic nursing program. Nurse Educ Today.

[3] Dunn KE, Osborne C, Rakes GC (2013). It’s not my fault: understanding nursing students’ causal attributions in pathophysiology. Nurse Educ Today.

[4] Elberson KL, Vance AR, Stephenson NL, Corbett RW (2001). Cooperative learning: a strategy for teaching pathophysiology to undergraduate nursing students. Nurse Educ.

[5] Branney J, Priego-Hernández J (2018). A mixed methods evaluation of team-based learning for applied pathophysiology in undergraduate nursing education. Nurse Educ Today.

[6] Alharbi M, Kuhn L, Morphet J (2022). The relationship between social media usage by undergraduate nursing students and development of their professional identity: a correlational study. Nurse Educ Today.

[7] Duke VJA, Anstey A, Carter S, Gosse N, Hutchens KM, Marsh JA (2017). Social media in nurse education: utilization and E-professionalism. Nurse Educ Today.

[8] Bressler T, Caceres BA (2018). Get a seat at the virtual table. Nurs Econ.

[9] Guidance on Using Social Media Responsibly. https://www.nmc.org.uk/globalassets/sitedocuments/nmc-publications/social-media-guidance.pdf.

[10] Luo H, Cai M, Cui Y (2021). Spread of misinformation in Social Networks: analysis based on Weibo Tweets. Secur Communication Networks.

[11] Peck JL (2014). Social media in nursing education: responsible integration for meaningful use. J Nurs Educ.

[12] Ross JG, Myers SM (2017). The current use of social media in undergraduate nursing education: a review of the literature. CIN: Computers Informatics Nursing.

[13] Jackson J, Gettings S, Metcalfe A. “The power of Twitter”: Using social media at a conference with nursing students. *Nurse Education Today* 2018, 68:188–191. 10.1016/j.nedt.2018.06.017: https://doi.org/10.1016/j.nedt.2018.06.017. 10.1016/j.nedt.2018.06.017 29945099

[14] Malik A, Heyman-Schrum C, Johri A (2019). Use of Twitter across educational settings: a review of the literature. Int J Educational Technol High Educ.

[15] Jones R, Kelsey J, Nelmes P, Chinn N, Chinn T, Proctor-Childs T (2016). Introducing Twitter as an assessed component of the undergraduate nursing curriculum: case study. J Adv Nurs.

[16] Stephens TM, Gunther ME (2016). Twitter, millennials, and nursing education research. Nurs Educ Perspect.

[17] Price AM, Devis K, LeMoine G, Crouch S, South N, Hossain R (2018). First year nursing students use of social media within education: results of a survey. Nurse Educ Today.

[18] Gazza EA (2019). Using Twitter to Engage Online RN-to-BSN students in Health Care Policy. J Nurs Educ.

[19] Mistry V (2011). Critical care training: using Twitter as a teaching tool. Br J Nurs.

[20] Junco R, Heiberger G, Loken E (2011). The effect of Twitter on college student engagement and grades. J Comput Assist Learn.

[21] Gonzalez SM, Gadbury-Amyot CC (2016). Using Twitter for Teaching and Learning in an oral and maxillofacial Radiology Course. J Dent Educ.

[22] Sinclair W, McLoughlin M, Warne T (2015). To Twitter to Woo: harnessing the power of social media (SoMe) in nurse education to enhance the student’s experience. Nurse Educ Pract.

[23] Ray A. Nursing students’ experiences on blogging in the classroom: Linking between ethics and pedagogy. 2014.

[24] Lowe B, Laffey D (2011). Is Twitter for the birds? Using Twitter to Enhance Student Learning in a marketing course. J Mark Educ.

[25] Scott N, Goode D (2020). The use of social media (some) as a learning tool in healthcare education: an integrative review of the literature. Nurse Educ Today.

[26] De Gagne JC, Yamane SS, Conklin JL, Chang J, Kang HS (2018). Social media use and cybercivility guidelines in US nursing schools: a review of websites. J Prof Nurs.

[27] Booth R, Connor S (2017). Meaningful use of Twitter in nursing education may improve student learning and should be considered as a viable educational tool to assist in the development of digital professionalism. Evid Based Nurs.

[28] Gagnon K (2015). Using twitter in health professional education: a case study. J Allied Health.

[29] Tubaishat A (2018). Student nurses’ perceptions of Facebook™ as an interactive learning platform in nursing education. Contemp Nurse.

[30] Warshawski S, Bar-Lev O, Barnoy S. Role of academic self-efficacy and social support on nursing students’ test anxiety. Nurse Educ. 2019;44(1):E6–e10. 10.1097/nne.0000000000000552. 10.1097/NNE.0000000000000552 29847355

[31] Sharp P, Ion R, Massey D (2018). Developing the social media presence of @NurseEducToday by using Twitter. Nurse Educ Today.

