# Supplementary material for: Effects of Twitter use on academic performance and satisfaction in a pathophysiology course among Omani nursing students: a quasi-experimental study
Source: BMC Nurs. 2023 Nov 21;22:439. doi: 10.1186/s12912-023-01609-x (PMC10662629; doi:10.1186/s12912-023-01609-x)
Supplement: Supplementary file 1 — Supplementary Material 1 [file 12912_2023_1609_MOESM1_ESM.docx]

**Effects of Twitter Use on Academic Performance and Satisfaction in a Pathophysiology Course Among Omani Nursing Students: A Quasi-Experimental Study**

1. **Demographic characteristics**
2. Before this year had you heard of twitter O Yes O No
3. Gender O Female O Male
4. Age
5. GPA
6. **Use of Twitter**
7. Did you have a Twitter account prior to this year? O Yes O No
8. On average, how many tweets do you send a week? O N/A

O 0 - 1 tweet O 2 – 3 tweets

O 4 – 5 tweets O 6+ tweets

1. On average, how many tweets did you send yesterday? O N/A

O 0 - 1 tweet O 2 – 3 tweets

O 4 – 5 tweets O 6+ tweets

1. Did you create an account for the pathophysiology course? O yes O No

(if yes, skip to question 11)

1. **Question-and-Answer session (Gonzalez and Gadbury-Amyot (2016))**

Answer the following questions with an X using the scale from strongly agree to strongly disagree (Select only one answer per question)

|  | Strongly agree | Agree | Neutral | Disagree | Strongly Disagree |
| --- | --- | --- | --- | --- | --- |
| 1. The question and answer sessions on Twitter were very helpful |  |  |  |  |  |
| 1. The use of Twitter for question and answer sessions had a positive effect on my overall grade |  |  |  |  |  |
| 1. I feel using Twitter for question and answer sessions improved my overall grade |  |  |  |  |  |
| 1. The twitter question and answer sessions improved accessibility to the instructor |  |  |  |  |  |
| 1. In the future I would enjoy using Twitter in other courses |  |  |  |  |  |
| 1. In the future I would enjoy using Twitter in the classroom for asking questions during lecture |  |  |  |  |  |

1. **Use of Twitter for the Pathophysiology course (Lowe and Laffey (2011))**

| Satisfaction | Strongly agree | Agree | Neutral | Disagree | Strongly disagree |
| --- | --- | --- | --- | --- | --- |
| 1. I believe that using web technologies such as Twitter is enjoyable |  |  |  |  |  |
| 1. Using Twitter increased my overall satisfaction with the course |  |  |  |  |  |
| 1. Using Twitter was one of the best parts of this course |  |  |  |  |  |
| 1. As a learning experience, using Twitter was more productive than listening to a lecture |  |  |  |  |  |
| 1. As a learning experience, using Twitter was more enjoyable than listening to a lecture |  |  |  |  |  |

1. Can you describe what aspects of using Twitter in the pathophysiology course you found beneficial or enjoyable?
2. Could you elaborate on any challenges or aspects of using Twitter in the pathophysiology course that you did not like or found unhelpful?
